# Recent Insights in Noninvasive Diagnostic for the Assessment of Kidney and Cardiovascular Outcome in Kidney Transplant Recipients

**DOI:** 10.3390/jcm13133778

**Published:** 2024-06-27

**Authors:** Peyman Falahat, Uta Scheidt, Daniel Pörner, Sebastian Schwab

**Affiliations:** Department of Internal Medicine I, Nephrology Section, University of Bonn, 53121 Bonn, Germany

**Keywords:** kidney transplantation, biomarker, MACEs

## Abstract

Kidney transplantation improves quality of life and prolongs survival of patients with end-stage kidney disease. However, kidney transplant recipients present a higher risk for cardiovascular events compared to the general population. Risk assessment for graft failure as well as cardiovascular events is still based on invasive procedures. Biomarkers in blood and urine, but also new diagnostic approaches like genetic or molecular testing, can be useful tools to monitor graft function and to identify patients of high cardiovascular risk. Many biomarkers have been introduced, whereas most of these biomarkers have not been implemented in clinical routine. Here, we discuss recent developments in biomarkers and diagnostic models in kidney transplant recipients. Because many factors impact graft function and cardiovascular risk, it is most likely that no biomarker will meet the highest demands and standards. We advocate to shift focus to the identification of patients benefitting from molecular and genetic testing as well as from analysis of more specific biomarkers instead of finding one biomarker fitting to all patients.

## 1. Introduction

In kidney transplant recipients (KTRs), clinicians mainly use serum creatinine and proteinuria to monitor graft function. Integration of these parameters and the detection of anti-HLA donor specific antibodies (DSAs) allows a risk assessment for long-term allograft failure. However, risk assessment still largely depends on histological findings such as interstitial fibrosis, tubular atrophy, glomerulitis, peritubular capillaritis, interstitial inflammation, tubulitis, and allograft glomerulopathy, since they have been found to be independent predictors for graft failure [1]. Therefore, most often, a decline in kidney function, an increase in proteinuria, or detection of donor-specific antibodies leads to kidney biopsy. Although a kidney biopsy is usually performed safely in transplant patients, it is an invasive procedure with serious risks. 

For the past 30 years, the Banff classification has been established as an international consensus classification by pathologists for the reporting of biopsies from kidney transplant recipients. Interestingly, in 2021 the Banff Minimally Invasive Diagnostics Working Group was established, aiming to evaluate the potential of minimally invasive biomarkers (e.g., donor-derived cell free DNA, blood and urine transcriptomics, und urinary chemokines) for integration into the Banff classification [2] as well as to distinguish between antibody-mediated rejection (AMR) and T-cell mediated rejection (TCMR) [3]. The latest version from 2021 implied non-DSA-biomarkers as screening-parameters to rule out rejection, directly diagnose, or confirm choices to perform biopsy [4]. The implementation of biomarkers into a histological classification further highlights the potential utility of biomarkers. 

Cardiovascular risk of patients after kidney transplantation remains to be higher compared to the general population [5]. It is essential to monitor and stratify cardiovascular risk in these patients, but determination of post-transplant cardiovascular risk is difficult and is currently depending on apparative and invasive diagnostic. Various biomarkers related to different organs and systems have been associated with cardiovascular risk and major adverse cardiovascular events (MACEs) in KTRs. 

Individual biomarkers are needed to identify individual and patient-specific risks for loss of graft function, but also to assess cardiovascular risk. Many novel biomarkers have been introduced lately and open up chances for wider use in clinical practice. Within this review, we will discuss heterogeneous biomarkers as well as new molecular diagnostic tools for kidney and cardiovascular outcome in KTRs. 

## 2. Assessment of Kidney Outcome after Kidney Transplantation

### 2.1. Biomarker for Glomerular and Tubular Function

The most commonly used biomarker to assess graft function is serum creatinine. An increase in serum creatinine is strongly associated with graft failure, but provides only a static model and is therefore less predictive than dynamic models. A recent study [6] proposed a dynamic, time-dependent model, called DynPG, which is based on six baseline values, i.e., serum creatinine at three months after transplantation, cardiovascular history, serum HLA class I-immunization levels previous to transplantation, recipient age, graft rank, and occurrence of acute rejection in the first year post-transplant. With any new serum creatinine measurement, a new DynPG score can be calculated, which allows a dynamic prediction of graft function. 

The score was validated externally in an observational study with 1637 patients up to 6 years after transplantation. Even though the learning and validation groups differed in patient and graft characteristics, the score provided good performance in predicting patient and graft survival [6]. 

A recently established marker for tubular atrophy and interstitial fibrosis (IF/TA) is urinary Dickkopf 3 (DKK3), a cysteine-enriched profibrotic glycoprotein released by stressed epithelial cells in the tubulus. It causes IF/TA by engaging a T-cell response and interacting with the Wnt signaling pathway. In acute kidney injury (AKI), DKK3 is well studied and is seen as a predictor of a kidney failure [7]. A recent study analyzed DKK3 in 122 kidney transplant recipients and documented a correlation between higher urinary DKK3 values and higher serum creatinine as well as higher albuminuria at 3 and 12 months after transplantation [8]. Therefore, DKK3 seems to be a potential predictor of graft function.

Chemokines, which circulate mainly in the blood but also in the urine, are extensively investigated as potential biomarkers. In particular, urinary chemokines increase before and during rejection [9], with chemokine interferon-inducible protein 9 (CXCL9) and chemokine interferon-inducible protein 10 (CXCL10) being the most feasible ones for prediction of graft function loss. In 80 kidney transplant recipients, urinary CXCL10 above a cut-off value of 84.73 pg/mL was predictive for graft rejection with a positive predictive value of 90% and a negative predictive value of 85% (AUC: 0.87) [10]. Both CXCL9 and CXCL10 were significantly elevated in 125 recipients with acute rejection [11]. Further, pretransplant CXCL10 levels higher than 150 pg/mL were associated with an increased risk of early and severe acute rejection and chronic allograft nephropathy [12]. 

Urinary neutrophil gelatinase-associated lipocalin (NGAL), a protein produced by activated neutrophils in the tubulus system, has been introduced as a valuable predictor for delayed graft function [13]. In 109 patients with stable graft function at the time of recruitment, NGAL levels were twice as high in kidney transplant recipients with a 10% decrease in eGFR over 1-year follow-up compared to those with stable or improving transplant function [14]. Nonetheless, establishing a cut-off value is challenging due to the use of different assays, which results in a lack of reproducibility. 

A less extensively studied biomarker is urinary kidney injury molecule-1 (uKIM-1), a transmembrane protein, with an immunoglobulin and mucin domain, whose expression is upregulated in damaged proximal tubules. Patients with higher urine levels (5.885 (4.420–7.913) vs. 4.605 (3.417–5.653) ng/mmol) on the first day after transplantation had a 23.5% higher risk of developing a delayed graft function [15]. A more recent study with 30 living donor and 85 deceased donor KTRs found no predictive capacity for KIM-1 levels in regard to delayed graft function. However, the authors demonstrated a correlation between the level of KIM-1 gene expression before reperfusion and the degree of interstitial fibrosis as detected by kidney biopsy [16].

### 2.2. Molecular Biomarkers

A human transcriptome is a complete set of RNA transcripts in a specific type of tissue. Transcriptome analysis is one of the most feasible candidates for classification of kidney allograft rejection. By measuring the gene expression of mRNA in biopsy samples of kidney or heart transplants, the Molecular Microscope Diagnostic System (MMDx) is the gold standard for transcriptome analysis in the diagnosis of AMR and TCMR [17]. The mRNA-pattern of a patient is compared to a set of over 8000 reference-specimens by an algorithm. Released by the Banff Molecular Diagnostics Working Group, the Banff-Human Organ Transplant (B-HOT) panel has been commercially available since 2019 and derives a gene list of 770 genes from peer-reviewed comprehensive microarray studies that were validated in biopsies from multiple organ transplants [18]. AlloMap, a blood-based gene expression profiling assay, is widely used and implemented in the monitoring of heart transplant recipients [19]. However, its use in kidney transplantation has so far produced controversial results, preventing a wider use [20,21]. Tests specific to kidney transplantation, such as the kSORT (Kidney Solid Organ Response Test), measure relative blood mRNA expression levels of 17 different genes associated with kidney allograft rejection or leukocyte activity [22]. The kSORT detects allograft failure at a high level (92% sensitivity and 93% specificity) but has no power in differentiating between TCMR and AMR.

Cell-free deoxyribonucleic acid (cfDNA) is a DNA fragment released into the blood by cells with rapid turnover. Donor-derived cell-free DNA (ddcfDNA) is cfDNA that comes from the transplanted kidney and is exogenous to the recipient. By measuring ddcfDNA, real-time [23] monitoring of graft function is possible. Urine and serum levels can reflect the status of graft injury [24]. Accuracy varies between studies, but is given up to 96% for antibody-mediated rejection in kidney transplant recipients [25]. A systematic review of diagnostic capacity of ddcfDNA in the detection of rejection in kidney transplantation found a pooled sensitivity and specificity of different tests of 0.84 (95% CI, 0.75–0.9) and 0.8 (95% CI, 0.74–0.84), respectively (AUC of 0.89) [26]. Newer data found an association between dd-cfDNA and both types of rejection, AMR and TCMR. Further, the higher the ddcfDNA levels were, the more active and severe the rejection was. In a multicentric study with 1134 patients, ddcfDNA increased the discriminative possibilities of detecting rejection significantly [27].

Other potential biomarkers include micro ribonucleic acids (microRNAs), which are short, noncoding RNAs associated with tissue fibrosis. These ribonucleotides are involved in nearly all biological processes and a dysfunction leads to impaired cell function and development of disease. Detection methods include quantitative real-time polymerase chain reaction or next-generation sequencing analysis in different fluids, such as blood cells, urine, or plasma [18]. Easy to obtain and specific for several biological processes, microRNAs levels differ in several types of injury. In transplant recipients from deceased donors, the urinary miR-146a levels increased to higher levels compared to recipients from living donors, indicating a specific ischemic reperfusion injury in the transplanted kidney [28]. 

Depending on the type of injury, increased expression levels of several microRNAs such as microRNA-223 and microRNA-142-3p in kidney graft biopsy and/or blood are associated with TCMR and, thus, may discriminate between TCMR and normal graft function [29].

Several other microRNAs have been proposed for application in TCMR and AMR (e.g., urinary microRNA-10a [30], microRNA99a [31], and microRNA-223 for TCMR or microRNA-142-5p for chronic AMR) [32]. However, they all have in common that sensitivity and specificity are limited.

Another proposed option as a monitoring tool in kidney transplantation is extracellular vesicles (EVs), which are bilayer lipid membranes containing exosomes or amphisomes that act as carriers in signal transmission between cells [33]. Any cell is capable of producing EVs, making it difficult to trace the origin of the EV [34]. Newer technologies, such as next-generation sequencing or mass spectrometry, can identify the contents and continue to reveal interesting aspects of EVs. In order to assess kidney function, EVs are collected from urine. Since urine composition is highly variable, conditions for sample collection and reporting of the results must be strictly normalized [35]. Collection and purification of EVs remains a challenge, as they must remain intact for analysis [36]. 

Park et al. reported that T-cell-derived urinary EVs might alter during TCMR indicating kidney tubular T-cell infiltration. IKEA (integrated kidney exosome assay), a diagnostic platform based on T-cell-derived urinary EVs, is used as a TMCR biomarker [37]. However, studies have shown varying numbers of EVs containing CD31, CD81, or urinary mRNA, correlating with cold ischemia duration, donor age, or reduced allograft blood flow [38,39]. 

**Figure 1:**
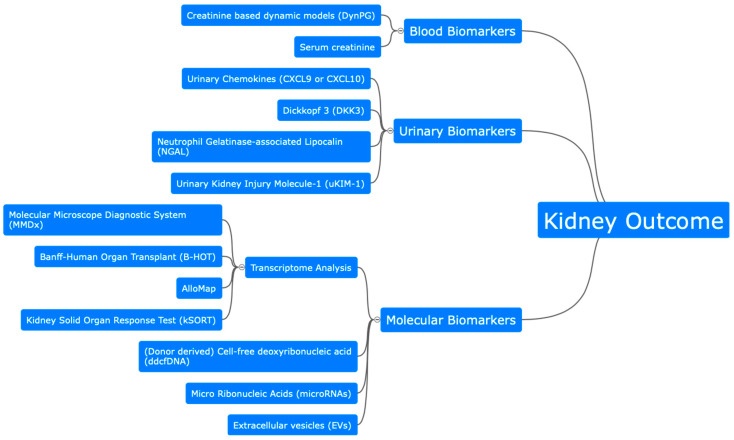
Graphical summary of possible diagnostic for estimation of kidney outcome after kidney transplantation.

As shown above, various biomarkers are being evaluated and developed for future clinical use. Dynamic models, such as DynPG, improve predictions by using multiple baseline values and allow updating with each new creatinine measurement. Other urinary parameters, such as DKK3, indicate tubular atrophy and interstitial fibrosis and correlate with worse kidney function post-transplantation. Urinary NGAL and KIM-1 have been shown to be promising markers for delayed graft function, though results for KIM-1 are inconsistent. Chemokines like CXCL9 and CXCL10 also show potential, particularly in predicting acute rejection with high sensitivity and specificity. Transcriptome analysis, especially using the Molecular Microscope Diagnostic System (MMDx) and gene panels like B-HOT, aids in diagnosing graft rejection. Most promising is donor-derived cell-free DNA (ddcfDNA) in blood, which allows dynamic monitoring of graft function. Lastly, microRNAs and extracellular vesicles (EVs) are being explored for their potential to monitor graft health and predict rejection, though challenges in standardization and sensitivity remain (Figure 1).

## 3. Biomarkers for Cardiovascular Risk in Kidney Transplant Recipients

### 3.1. Serum, Immunological, and Metabolic Biomarker 

Albuminuria is predictive for cardiovascular outcome in patients with cardiovascular disease as well as in patients with end-stage kidney disease [40] and can be predictive for mortality and increased risk of graft loss in KTRs [41,42]. A recent study showed that albuminuria is predictive for MACEs in KTRs even after adjustment for cardiovascular risk factors like diabetes, cholesterol levels, age, or sex [43]. However, this study was a single-center study with several limitations like a limited applicability to non-Caucasian patients and a rather small sample size. 

Beta trace protein (BTP) is a serum biomarker known to be accurate for the assessment of residual kidney function in patients with end-stage kidney disease [44,45] and has previously been associated with post-transplant MACEs. In opposition to that, other markers of kidney function like cystatin C did not associate with posttransplant MACEs [46]. 

Not only kidney, but also cardiac biomarkers are associated with cardiovascular risk in KTRs. Amino-terminal pro-B-type natriuretic peptide (NT-proBNP) is a hallmark biomarker for heart failure and left ventricular dysfunction [47,48]. It is secreted as response to elevated wall tension in the left ventricle [49]. Two recent studies have also shown that NT-proBNP is associated with cardiovascular outcome in KTRs. The difference within these two studies was the time point of measurement, as one study measured NT-proBNP before and the other one after transplantation. Pretransplant NT-proBNP was significantly associated with MACEs [50], whereas post-transplant NT-proBNP was associated significantly with heart failure and all-cause mortality [51]. In the latter study, Troponin T also showed an association with MACEs. This is in line with a previous study, which demonstrated an association between Troponin T and all-cause mortality in KTRs [52]. 

Inflammatory processes are key players in the development of atherosclerosis and MACEs [53]. Inflammatory environments already develop before transplantation, but continue to be present after transplantation. Although not within the focus of the review, it is important to note that inflammatory biomarkers like IL-6 and CRP have been found to be independent risk factors for cardiac events and death in KTRs [54,55,56]. 

In the complex cascade of inflammation involved in the development of atherosclerosis, it has been shown further that human leukocyte antigen (HLA) class II is expressed in inflammatory cells within the atherosclerotic plaque as a result of induction via the Interferon-γ pathway [57,58]. 

A single-center study in 2020 showed that serum pretransplant HLA class II antibodies are associated with MACEs, a cardiovascular and all-cause mortality. The authors also analyzed for HLA DR 1, an HLA class II molecule, and demonstrated that KTRs who received an organ from a donor who was HLA DR I-positive had a higher risk for MACEs and all-cause mortality. In contrast, HLA-DR1 positivity in KTRs was not associated with MACEs or all-cause mortality. Most of the patients, who were HLA II-positive, were women or had previous kidney transplantations [59]. An earlier study from 2015 showed that HLA antibodies in general, and especially donor-specific HLA antibodies, are highly associated with MACEs in KTRs [60].

Accumulation of calcium salts leads to arteriosclerotic calcification. Various biomarkers of calcium metabolism play an important role in predicting cardiovascular risk in KTRs. One prominent example is serum osteoprotegerin (OPG) [61,62,63]. It is a member of the tumor necrosis factor family and a soluble decoy receptor for receptor activators of nuclear factor kappa-B ligand (RANKL). With its role in the RANKL-pathway, it is involved in regulating osteoclast function and inhibiting bone resorption, which leads to prevention of vascular calcification [64]. It seems counterintuitive that elevated serum OPG levels are associated with MACEs in non-KTRs [65] as well as after kidney transplantation, regardless of the time of measurement after transplantation [61,62]. 

Another biomarker associated with calcium–phosphorus metabolism is calciprotein particles (CPPs), which are blood-borne insoluble nanoparticles containing supraphysiological levels of phosphorus and calcium [66]. These may have cytotoxic effects and can lead to a calcification off vessels. Primary CPPs transform into secondary CPPs, which are more harmful [67]. A test, called T50, reflects half the transformation time from primary to secondary CPPs. It uses three-dimensional cross-correlation dynamic light scattering to detect the transformation from primary CPPs, containing amorphous calcium phosphate, to the secondary, spindle-shaped CPPs [68]. A shortened T50 indicates a greater predisposition to the formation of secondary CPPs, leading to an increased likelihood of vascular calcification [69]. It is not surprising that a number of studies have shown that reduced T50 is associated with cardiovascular and all-cause mortality, as well as the risk of graft failure [69,70,71]. Fetuin-A, a calcification inhibitor and another component of CCPs, is also associated with cardiovascular death [72].

### 3.2. Insights from Genetic Analysis

Genetic factors influence cardiovascular risk of KTRs. A study from 2019 investigated whether a genetic risk score based on 27 single nucleotide polymorphisms (SNPs) was able to predict cardiovascular disease in KTRs [73]. These 27 SNPs were tested after blood purification to predict cardiovascular events in a community-based cohort in a previous study [74]. However, it has also been shown that not every single SNP has influence on predicting cardiovascular outcome. Therefore, a slightly different selection of SNPs should be chosen for a prospective genetic risk score focused on KTRs. 

**Figure 2:**
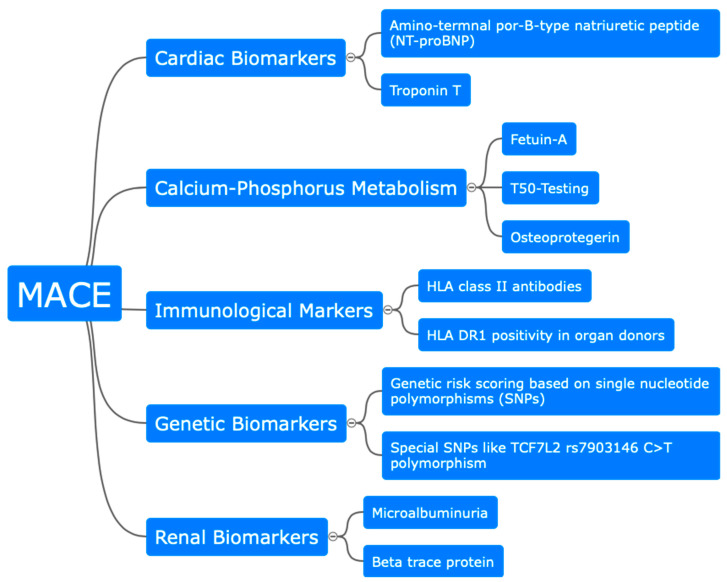
Graphical summary of possible diagnostics for estimation of cardiovascular outcome after kidney transplantation.

One SNP was investigated in a single-center study showing that the TCF7L2 rs7903146 C>T polymorphism, which is known to be associated with diabetes mellitus, is associated with post-transplant MACEs [75]. Because it is established that this polymorphism is associated with the cardiovascular risk factor diabetes mellitus, all KTRs with pre- or post-transplant diabetes were excluded. However, in both studies, mainly Caucasian participants were included and it is likely that genetic factors will differ in other ethnicities (Figure 2).

Certainly, with increasing availability, genetic testing will reveal more insight in genetic variants at risk for undesirable kidney and cardiovascular outcome.

A variety of factors influence the cardiovascular outcome in KTRs and, as a result, a variety of biomarkers have shown interesting results in predicting the very same. However, many of the studies summarized here have significant limitations such as the single-center nature of many of the studies, the lack of adjustment for confounding factors, or the lack of comparison with a control group.

## 4. Conclusions

Within this review, we discussed several different biomarkers and molecular approaches that have been associated with graft failure as well as cardiovascular risk following kidney transplantation. We discussed biomarkers in static and dynamic models and highlighted the potential value of molecular analysis and genetic testing for polymorphisms.

It is obvious that a multitude of factors have an impact on kidney, cardiovascular, and overall outcome of kidney transplant recipients. It is therefore unlikely that a single biomarker will be established to identify and guide personal risk for undesired outcomes.

One of the least considered factors is sex-related differences in biomarker expression. Although this was beyond the scope of the review, to the best of our knowledge, there are very limited data for KTRs and we can only speculate about the impact of gender-related differences on the introduced biomarkers. The Kidney Disease Improving Global Outcomes (KDIGO) practical guidelines states that female sex is among the susceptibility factors for nonspecific acute kidney injury [76]. Most likely, sex-related differences in noninvasive diagnostics in KTRs also exist and are so far insufficiently addressed. In light of this, age- and ethnicity-dependent heterogeneity amongst biomarker expression also remains to be elucidated in future studies.

In our opinion, serum creatinine, proteinuria, allograft histology, and DSA will continue to be the basis for risk stratification. We are well aware that to date, molecular and genetic testing is cost-intensive and not available in many regions of the world, but these diagnostic tools will become more affordable. Future research should focus more on identification of patients that will benefit from molecular and genetic testing. This could pave the way to a more personalized and tailored risk stratification approach.
